# Adoptive neoantigen-reactive T cell therapy: improvement strategies and current clinical researches

**DOI:** 10.1186/s40364-023-00478-5

**Published:** 2023-04-17

**Authors:** Ruichen Huang, Bi Zhao, Shi Hu, Qian Zhang, Xiaoping Su, Wei Zhang

**Affiliations:** 1 Department of Respiratory and Critical Care Medicine, the First Affiliated Hospital of Second Military Medical University, Shanghai, 200433 People’s Republic of China; 2 Department of Biophysics, College of Basic Medical Sciences, Second Military Medical University, 800 Xiangyin Road, Shanghai, 200433 People’s Republic of China; 3 National Key Laboratory of Medical Immunology, Institute of Immunology, Second Military Medical University, 800 Xiangyin Road, Shanghai, 200433 People’s Republic of China; 4 School of Basic Medicine, Wenzhou Medical University, Wenzhou, 325000 People’s Republic of China

**Keywords:** Neoantigen-reactive T cell, Adoptive cell therapy, Immunotherapy, Cancer

## Abstract

Neoantigens generated by non-synonymous mutations of tumor genes can induce activation of neoantigen-reactive T (NRT) cells which have the ability to resist the growth of tumors expressing specific neoantigens. Immunotherapy based on NRT cells has made preeminent achievements in melanoma and other solid tumors. The process of manufacturing NRT cells includes identification of neoantigens, preparation of neoantigen expression vectors or peptides, induction and activation of NRT cells, and analysis of functions and phenotypes. Numerous improvement strategies have been proposed to enhance the potency of NRT cells by engineering TCR, promoting infiltration of T cells and overcoming immunosuppressive factors in the tumor microenvironment. In this review, we outline the improvement of the preparation and the function assessment of NRT cells, and discuss the current status of clinical trials related to NRT cell immunotherapy.

## Background

Recently, groundbreaking immunotherapies have revolutionized the schemes of cancer treatment. Conventional immunotherapies include immune checkpoint inhibitors (ICIs), adoptive cell therapy (ACT) and cancer vaccines, all of which improve the capability of immune system of recognizing and attacking cancer cells [1, 2]. However, due to the heterogeneity of tumors, Immunotherapy targeting single antigen may also result in the generation of target-irrelevant tumor cell clones and tumor immune escape, which has been reviewed in reference [3]. Therefore, it is urgent to develop multi-targeted immunotherapy. The term “neoantigen” means a new epitope of autoantigens generated by somatic non-synonymous mutations [4]. And cancer neoantigens will be generated by this kind of DNA mutations accumulated in tumor cells [5]. These antigens have tumor specificity and are absolutely absent in normal cells; they also possess the ability to stimulate autoimmune response and are not subject to central immune tolerance [6]. Targeting multiple neoantigens can be a significant measure to deal with the challenge of tumor immune escape. Since adoptive therapy with tumor infiltrating lymphocytes (TILs) emerged in the 1980s, neoantigens have been found as the major targets of TILs to exert specific antitumor function. Researches showed that these neoantigens can induce neoantigen-specific T cells, also called “neoantigen-reactive T cells” or “NRT” cells. Researches on NRT-based immunotherapies, including neoantigen vaccine and NRT cell adoptive therapy, have made remarkable achievements in melanoma and other solid tumors [7, 8]. The common point of these therapies is to recognize and kill neoplastic cells with autologous or heterologous NRT cells. However, Zhuting Hu et al. noted that neoantigen vaccine cannot induce adaptive immunity if not combined with appropriate adjuvants in the review of [6]. Even after activation, this vaccine still upregulates the immunosuppressive signaling of cancer, leading to the formation of suppressive tumor microenvironment (TME) [9]. What is more, weak immune induction is the most obvious defect of neoantigen vaccine in the treatment of advanced solid tumors. By contrast, a recent review has revealed that NRT cells can directly infiltrate into tumors after cultivation, and overcome the inhibition from TME by genetic modification of signal molecules [10]. For these reasons, developing NRT cell adoptive therapy can be a more effective method in treating solid tumors. This review focuses on the development history, preparation process, and preclinical as well as clinical researches of NRT cell therapy. It also explores the methods to enhance the anti-tumor effect of NRT cells.

### Development history of NRT cell therapy

In the 1980s, De Plaen E. et al. first explored a neoantigen deriving from a single nucleotide variant which could be recognized by cytolytic T cells [11]. Subsequently, numerous cancer-related mutations that can be recognized by T cells were identified, including tumor associated antigens (TAAs), tumor specific antigens (TSAs), and cancer or testis antigens [12–16]. Among them, TSAs, especially neoantigens, are considered as the optimal tumor targets because they are never expressed in normal tissues and have a low probability of inducing tolerance. In a recent review, this group of antigens were divided into three types: Guarding neoantigens can induce antitumor immune response independently; restrained neoantigens have immune checkpoint-dependent immunogenicity; ignored neoantigens lack spontaneous immunogenicity [17]. The majority of neoantigens belong to the “ignored neoantigen” type, regarded as “the reserve of neoantigens”, which can be prepared as vaccine to induce autologous NRT cells [17]. In 2004, Rosenberg and his colleagues completed the first case of adoptive cell therapy, which showed that the tumor in metastatic lesions of patients with malignant melanoma regressed completely after adoptive transfer of TILs [18]. In another study, they also demonstrated that this therapy with two identified neoantigens can promote tumor infiltration of NRT cells and prolong their persistence [19]. The emergence of the next-generation sequencing technology has brought a new dawn for screening tumor neoantigens. This technique, combined with major histocompatibility complex (MHC) binding prediction approach based on silico algorithms, facilitates the selection of optimal missense genes and has become the mainstream method in neoantigen-identification [20, 21]. Patrick A. Ott et al. observed that neoantigen vaccine, another neoantigen-based immunotherapy, induces significant anti-tumor immune response in melanoma patients [22]. This therapy provides another reasonable, safe and durable anti-tumor method in a more individualized mode, but it has failed to achieve clinical benefits in a wider spectrum of cancer patients, which is addressed in the review of [23]. However, the combined treatment of neoantigen vaccines and immune checkpoint inhibitors at least partly provides a reference scheme for enhancing the clinical response of NRT cell treatment [24]. Currently, The focus of NRT cell therapy has shifted from melanoma to other solid tumors [25–29]. However, the efficacy of this therapy in solid tumors is limited, which is related to tumor immune escape, immune cell exhaustion or dysfunction, and immunosuppressive state of the tumor microenvironment (TME). Current NRT therapy mainly stems from improvement on TILs adoptive therapy. Neoantigen vaccines, adoptive transfer of NRT cells, TCR-engineered T cells and chimeric receptor T cell therapy have gradually emerged in clinical individualized antitumor treatment. Encouraging results from clinical studies highlight the importance of NRT cells in antitumor immunity. However, because few researches have studied adopting NRT cell therapy, very limited information of how to increase the efficacy of NRT cells adoptive therapy can be obtained from completed clinical trials hitherto. More endeavors are therefore needed to dissect the relationship between tumor immunity, neoantigens, and immune cells.

### Introduction of neoantigen detecting platforms

**Fig. 1:**
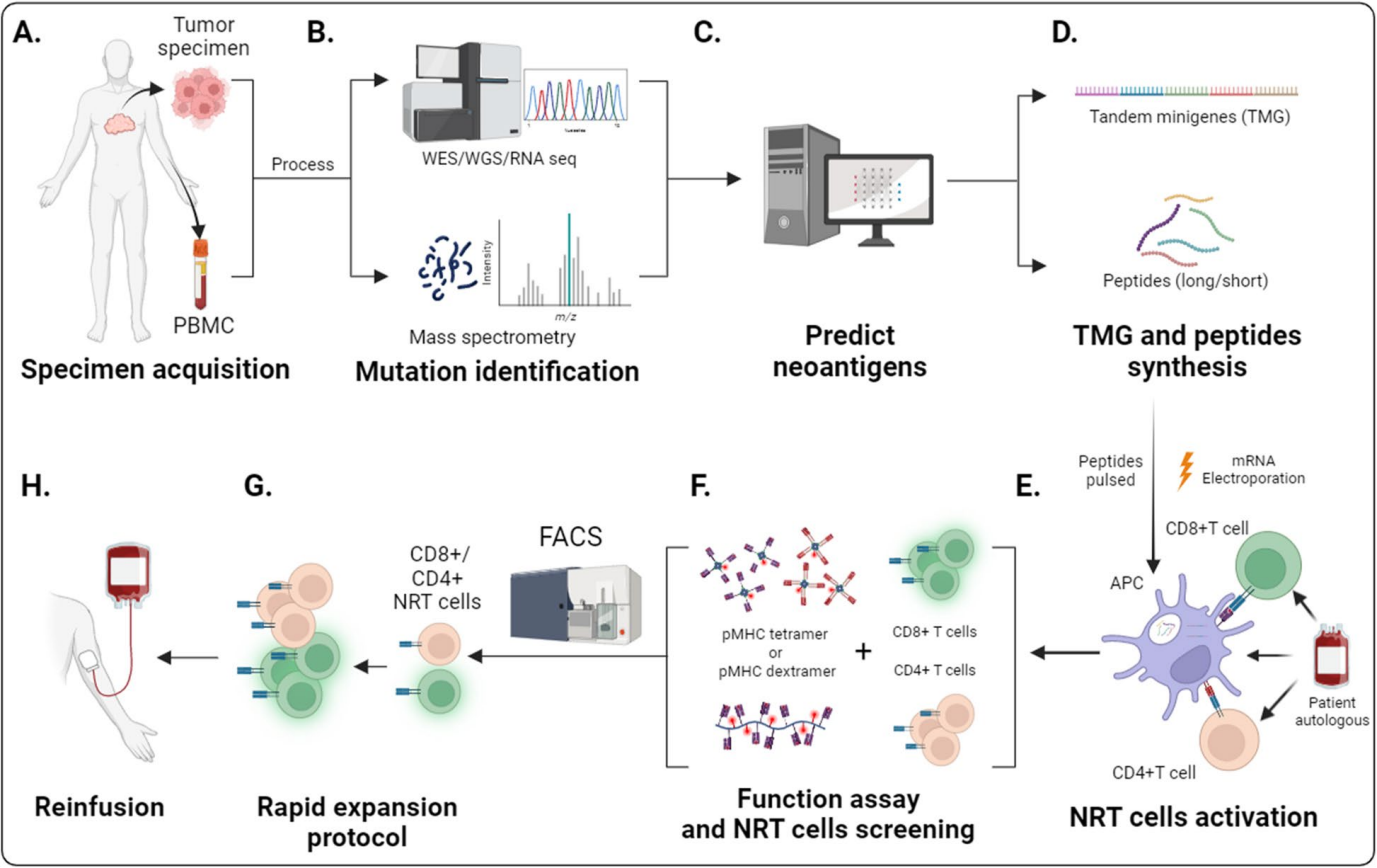
Process of NRT cell manufacturing and adoptive therapy. NRT cells are manufactured via the following steps: **A** acquisition and cultivation of tumor specimens and peripheral blood mononuclear cells; **B** mutation identification with WES/WGS/RNA sequencing (seq), potential antigen detection with mass spectrometry; **C** neoantigen prediction; **D** design and synthesis of neoantigen-encoding mRNA in tandem minigene configuration or neoantigen peptides; **E** pulsing DCs directly with peptides, or transfection of neoantigen-encoding mRNA into DCs by electroporation, followed by the co-incubation of neoantigen-loaded DCs and PBMC-derived T cells, **F** flow cytometry-based neoantigen-specific T cell sorting; **G** rapid expansion protocol (REP) of NRT cells, **H** reinfusion of NRT cells into patients or mouse model

**Fig. 2:**
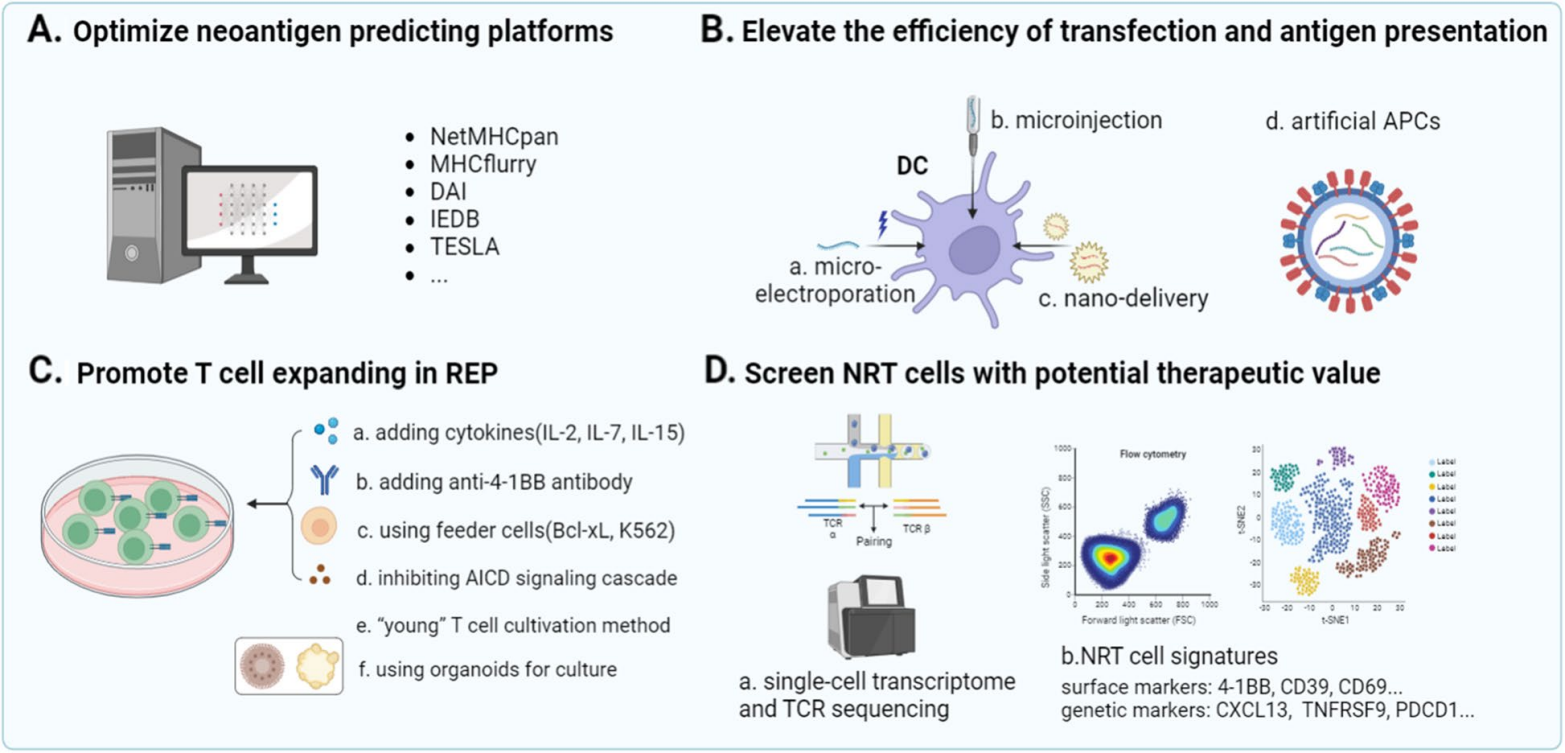
Feasible improvement for the manufacture of NRT cells. **A** Optimize neoantigen predicting platforms to promote the efficiency and accuracy of prediction. **B** Micro-electroporation (a), microinjection (b) and nano-delivery c can be applied to elevate transfection efficiency. Artificial APCs can be used to increase the efficiency of antigen presentation. **C** Promote NRT cell expansion in rapid expanding protocol (REP) through adding cytokines (IL-2, IL-7 and IL-15) (a) or anti-4-1BB antibody (b), using feeder cells (Bcl-xL, K562) (c), inhibiting AICD signaling cascade (d), or adopting the culture method of “young” T cells (e) or organoids (f). **D** Screen NRT cells with surface or genetic markers via single-cell transcriptome and TCR sequencing

Unlike overexpressed or abnormally expressed tumor-associated antigens, neoantigens are absent in the normal human genome [30]. The high-throughput sequencing technology and algorithmic prediction platforms render neoantigen identification more rapid and accurate (Fig. 1B, C) [31]. As for high-throughput sequencing, whole exome sequencing (WES) becomes the keystone of neoantigen identification [32]. Besides, mass spectrometry technology provides a large number of peptide data for training of MHC prediction platforms [33] (Fig. 1B). As for algorithmic prediction platforms, machine learning and artificial intelligence platforms (Fig. 2A) help to precisely predict potential MHC binding epitopes and MHC-peptide binding affinity based on sequencing outcomes. Some common prediction software, algorithms, and databases are listed in Table 1. Mutation screening is the first step in neoantigen identification. Mutation calling tools include Burrows-Wheeler Alignment tool (BWA), ANNOVAR, MuTect, SomaticSniper, VarScan, and FusionCatcher [34–38]. Differences between mutant sequence and wild sequences can be identified using the differential agretopic index (DAI) [39]. It is necessary to predict MHC binding ligands and binding affinity to determine whether mutations can form neoantigens. Published software represented by NetMHCpan, MHCflurry, HLAthena, MHCnuggets and ProGeo-Neo shows favorable outcomes in neoantigen prediction [40–44]. Immune Epitope Database (IEDB) is a primary epitope database and nealy all the prediction tools use the data from it [45]. The company of this database recently developed TCRMatch, which can identify T cell epitopes with unknown specificity based on optimized T cell epitope data of IEDB [46]. However, the data of IEDB are mostly from virus resources, which lead to the deviation of cancer neoantigen prediction. Novel database-Tumor Neoantigen Selection Alliance (TESLA)-based on tumor sequencing data will improve the precision of tumor neoantigen prediction [47]. The platforms widely applied in predicting MHC ligands are trained on neoepitope prediction with the data from literature or online databases. Each platform has the limitations of prediction objects and methods, while collaboration of multiple platforms will improve the specificity and accuracy. It is reasonable to prospect that these techniques will help to solve the difficulty in choosing the optimal neoantigen to activate antitumor NRT cells, which may be conducive to the efficacy improvement of immunotherapy.

**Table 1:** Common platforms and algorithms for neoantigen prediction

**Tools Name**	Function	Tool type	Ref
Burrows-Wheeler Alignment tool (BWA)	Alignment tool for mutation identification	software	[34]
ANNOVAR	Mutation identification and functional annotation	software	[35]
MuTect,SomaticSniper	Mutation calling and screening	software	[36, 37]
VarScan	Mutations and copy number alterations calling	software	[38]
FusionCatcher	Fusion gene mutation identification	software	http://code.google.com/p/fusioncatcher/
NetMHCpan/NetMHCpanII, MHCflurry	MHC-I/II binding affinity prediction	software	[41, 48–50]
MixMHCpred score, HLAthena	MHC-I binding ligands prediction	algorithm	[42, 51]
MHCnuggets	MHC-I or MHC-II binding ligands prediction	software	[43]
ProGeo-Neo	Mutation calling, MHC-I and MHC-II binding affinity and binding ligands prediction	software	[44]
Differential agretopic index(DAI)	Difference identification between mutant and wild sequences	algorithm	[39]
NetCTLpan	T cell epitope and MHC-I binding ligands prediction	software	[52]
TCRMatch	T cell epitope prediction	software	[46]
PyClone	Clonal population prediction	algorithm	[53]
MuPeXI	Neoantigen Immunogenicity identification	software	[54]
Immune Epitope Database (IEDB)	Epitope data	database	[45]
Tumor Neoantigen Selection Alliance(TESLA)	Epitope and sequencing data	database	[47]

### Process of NRT cell induction

The major purpose of predicting neoantigens precisely is to induce the immune response of NRT cells, which is the critical element for antitumor immunotherapy. To induce NRT cells, the wide accepted protocols mainly include: specimen acquisition and isolation (Fig. 1A), identification of non-synonymous mutation through WES/WGS/RNA sequencing (seq), and detection of potential antigens with mass spectrometry (Fig. 1B), neoantigen prediction utilizing bioinformation technology (Fig. 1C), design and synthesis of neoantigen-encoding mRNA in tandem minigene configuration or neoantigen peptides (Fig. 1D), pulsing DCs directly with peptides, or transfection of neoantigen-encoding mRNA into DCs by electroporation, followed by the co-incubation of neoantigen-loaded DCs and PBMC-derived T cells (Fig. 1E), NRT cell functional assay and sorting through flow cytometry (Fig. 1F), rapid expansion protocol (REP) of NRT cells (Fig. 1G) before reinfusion into patients or mouse model (Fig. 1H) [55, 56]. Then, efficacy assessments of NRT cell adoptive therapy will be performed. In this part, we will introduce the improvement strategies of NRT cell induction specifically.

Ameliorated technologies, such as microinjection, micro-electroporation and nano-delivery, should be taken into account to elevate transfection efficiency (Fig. 2B a, b, c) [57, 58]. Because of the limited capacity of antigen presentation and long duration of the induction process when using autologous DCs, modified strategies of allogeneic APCs have been put forward (Fig. 2B d). Synthetic APCs, including magnetic and polymer compound beads covered with anti-CD3/CD28 antibody and HLA-Ig, also can be used to activate NRT cells [59, 60]. Nanoparticle-based artificial antigen presenting cell can mimic DCs to effectively prime and expand T cells. The following are some ways whereby this APC can be engineered: adding co-receptors, synthesizing nanoparticles coated with molecule-labeled DC membrane and T cell targeted antigens, modifying the shape of nanoparticles and endowing anti-phagocytosis ability [61–64]. Introduction of IL-2, and low-level IL-7 and IL-15 can be a time-saving method for NRT cell priming in expanding process, and these two cytokines promote the formation of the effector phenotype and the central memory phenotype while IL-15 additionally induces the stem cell memory phenotype of T cells (Fig. 2C a) [65, 66]. Cultivating TILs with agonistic CD137 (4-1BB) monoclonal antibodies increases frequency of CD8 + TILs, as well as amplification rate and quantity of T cell subclone types (Fig. 2C b) [67]. The “feeder cell”, including immortalized B cells and K562 cells, can be modified to express signals for T cell proliferation, which wins the favor of researchers in the REP process(Fig. 2C c) [68, 69]. In addition, inhibiting AICD signaling cascade and preventing the aging of T cells (“young” T cultivation method) in the process of REP will enhance the activity and prolong the persistence of adoptively transferred T cells (Fig. 2C d, e) [70, 71]. Another strategy is to cultivate NRT cells with tumor organoids in a personalized manner (Fig. 2C f) [72]. This patient-specific cell culture method develops a platform for better exploring the interaction between T cells, tumor cells and other immune cells from native environment. The study of NRT cells based on multi-omics analysis has confirmed its feasibility [73]. Generally, a more efficient inducing process of NRT cells has important implications for NRT cell therapy. The above-mentioned strategies seek to improve the efficiency of vector transduction, and promote the amplification and activation of T cells.

### Detection of neoantigen-reactive T cell populations

Florian Kast et al. reviewed that using pMHC tetramer and dextramer binding assay based on flow cytometry can strengthen the binding forces between single pMHC and TCR, and elevate the efficiency of NRT cells screening (Fig. 1F) [4]. However, due to its low sensitivity, this technique cannot detect rare T cell clones containing NRT cells. Single-cell transcriptome and TCR sequencing play essential roles in the discovery of novel tumor-reactive T cell subclones, and further aid in the analysis and filteration of potential NRT cells within these subclones (Fig. 2E). Currently, the most common single-cell sequencing method of T cells mainly adopts microwell or microfluidic technology [74, 75]. Then, reverse transcription and PCR amplification are performed before transcriptomic and TCR sequencing. The sequencing data can be integrated and analyzed to identify T cell subclones and reconstruct TCR chains for pairing TCR with specific T cell clones [75, 76]. With the support of bioinformatic analysis, it is more convenient to find potential therapeutic targets and novel biomarkers with prognostic value, which facilitates efficacy evaluation of immunotherapies tailored to individuals. The feasibility of this novel detecting method has been demonstrated in several studies, and NRT cell populations have been successfully identified and isolated [77, 78]. The latest research also revealed that NeoTCR signatures can be used to identify specific antitumor NRT cells via single-cell transcriptome and single-cell TCR sequencing [79].

### The value of screening signatures in NRT cell identification

Currently accepted surface markers of NRT cell include CTLA-4, PD-1, LAG-3, TIM-3 and TIGIT. 4-1BB/CD137 is transiently expressed on T cells, which is regarded as specific activating signature of NRT cells and extensively used in NRT cell population screening [80, 81]. High frequency of CD39 + tumor reactive T cells is relevant to better prognosis in cancer patients [82]. NRT cells of stem-like double negative (CD39- CD69-) phenotype show stronger antitumor activity and longer persistence despite the rarity of these cells [83]. In addition, other novel NRT cell surface markers have been identified, including CXCL13 and CD200 [84–86]. Particularly, the expression of CXCL13 is significantly different between NRT cells and bystander T cells. Common gene signatures include PDCD1, ENTPD1, LAG3, TIGHT, TNFRSF9, HAVCR2, BATF, GZMA/B/K, IFNA/B/G and CXCL13 gene [79, 84]. In order to elevate sensitivity and specificity of NRT cell screening, a combination of surface markers and transcriptome markers is recommended for identifying NRT cells. This approach not only facilitates the cell screening process, but also circumvents the influence of functional assays on the viability of the cells.

### Feasible engineering strategies for NRT cells

**Fig. 3:**
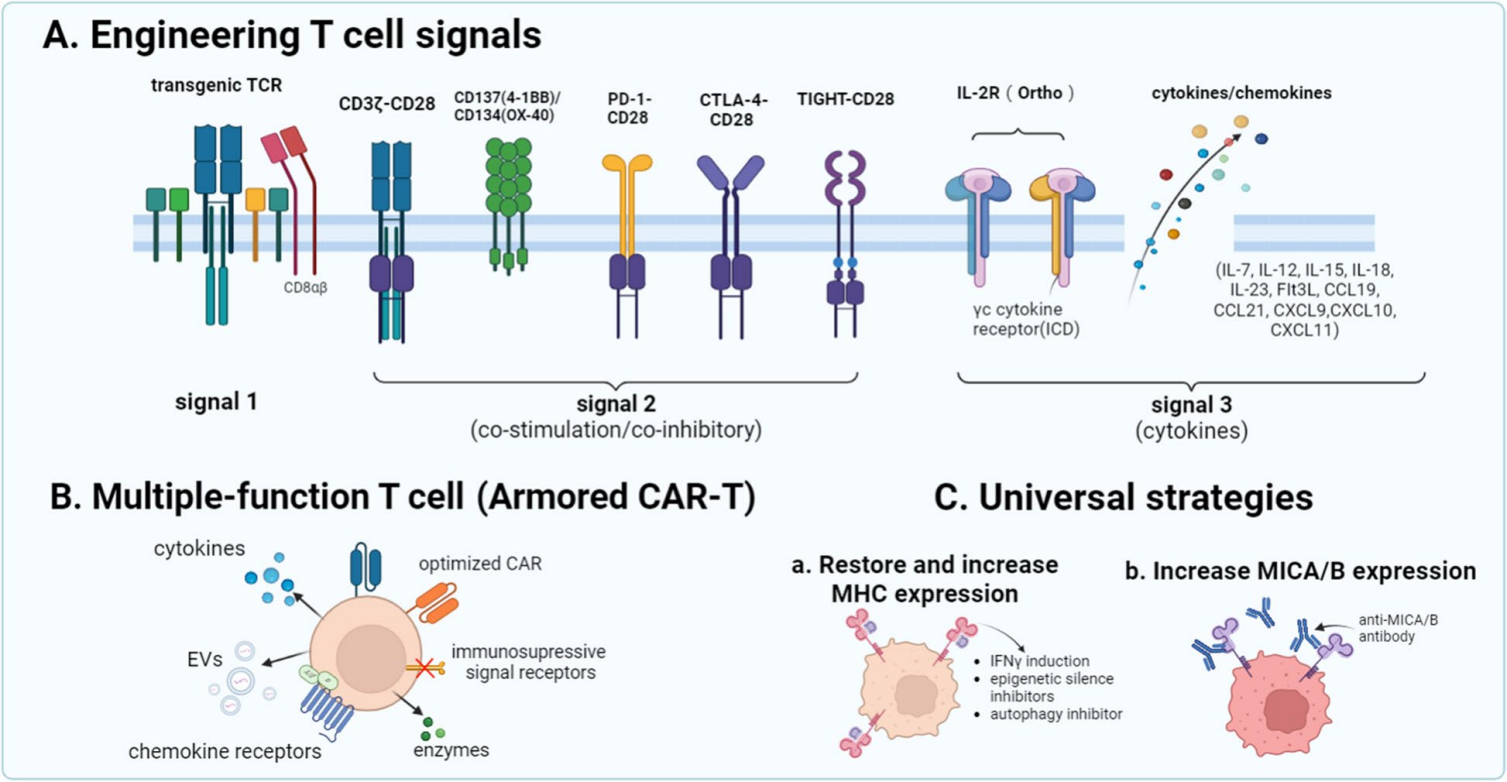
Feasible strategies for the improvement of T cell antitumor function. **A** Engineer T cell signals. Signal 1: edit TCR genes or make TCR and CD8 αβ co-expression. Signal 2: join CD28 to CD3ζ combined with 4-1BB or OX40 to enhance activation signals, construct the chimeric switch receptor (e. g., CD28 linked to PD-1,CTLA-4 and TIGHT) to reverse inhibitory signals. Signal 3: modify cytokine receptors (e. g., IL-2 orthogonal receptor) and increase the expression of autologous or heterologous cytokines or chemokines. **B** Produce multiple-function T cells: optimize CARs, secrete cytokines and enzymes, release extracellular vesicles containing RNAs, express multiple chemokine receptors, and modify immunosuppressive signal receptors. **C** Universal strategies to restore and increase the expression of MHC (inducing IFNγ production, using epigenetic silence or autophagy inhibitors (a)) and MICA/B (anti-MICA/B antibody (b))

The process of TCR recognizing and binding to MHC molecules is of great importance for T cells to perform antitumor function. The challenges are how to make T cells recognize tumor cells more effectively, how to enhance TCR function without increasing toxicity, and how to counteract the problems of T cell exhaustion as well as dysfunction. Therefore, we summarize some feasible engineering strategies for NRT cells to address these issues (Fig. 3A). Currently, the three most common engineering objects are TCR signals, co-stimulated signals and cytokines of T cells. Using transgenic TCR, co-expressing CD8 αβ with TCR and upregulating adhesion molecules can enhance MHC-TCR binding avidity and elevate signal-transducing efficiency [87–89]. Besides, engineering co-stimulatory signals is proposed to prolong T cell persistence and enhance anti-tumor activity, which can be achieved by coupling T cell activating signals (CD3ζ) with co-stimulating signals (CD28, OX40, 4-1BB), or using chimeric switch receptors which link exodomain of CTLA-4, PD-1 or TIGHT to intradomain of CD28 [90–95]. In addition, engineering cytokine receptors represented by orthogonal IL-2 has been found to enhance T cell antitumor function while attenuating the side effects caused by cytokine pleiotropy [96–98]. And T cells engineered to secrete additional cytokines (IL-7/12/15/18/23 and Flt3L) or chemokines (CXCL9/10/11 and CCL19/21) also show enhancement of activity and function [99–109]. These strategies improve the function of autologous tumor-reactive T cells, promote their phenotype switching, and, meanwhile, recruit other immune cells (such as NK cells and DCs) to exert antitumor effects. The abovementioned engineering strategies are used in CAR-T cells. CAR-T cells can also be designed to release enzymes, express multiple immunomodulators, deliver endogenous RNA, maximize the diversity of functions and minimize “off-target” toxicity, which are summarized as comprehensive strategies of armored CAR-T (Fig. 3B). These strategies have been reviewed in [110–113]. Engineering strategies of TCR T cells can also draw on this idea to extend persistence, promote homing and penetration into tumors, and enable T cells to target tumor cells and activate multiple immune cells simultaneously. Overall, the above researches highlight the necessity of T cell signaling in priming, proliferation and exerting function. Engineering strategies for receptors and cytokines expressed by T cells help to improve the persistence and antitumor function of T cells. We propose that these T cell modification methodologies can be also used to improve the antitumor activity as well as persistence of NRT cells, and promote infiltration of immune cells into solid tumors to limit their growth more effectively.

### Universal strategies for NRT cell therapeutic enhancement

MHC-TCR recognition pattern is the primary mechanism of NRT cell therapy. Under certain circumstances, however, tumor escape will occur when classical MHC molecules are downregulated or lose expression, which may be caused by gene reconstruction or mutation and deletion of functional components, loss of transcription factor, epigenetic silence, and pre-or post-transcriptional inhibitory regulation [114, 115]. Deficient expression of MHC also leads to dysfunction in the neoantigen presentation process [116]. This phenomenon will eventually affect the ability of T cells to recognize tumor cells. Since traditional TCR engineered T cells have MHC -restriction, modification of autologous T cells can be cumbersome and expensive. It is a tendency to use universal methods to enhance the antitumor function of T cells (Fig. 3C). These strategies can be used as auxiliary means to improve the efficacy of NRT cell therapy. The first strategy is to restore and increase expression of MHC molecules (Fig. 3C a). Previous researches have shown that IFN-γ can increase MHC expression [117]. Adopting epigenetic silence inhibitors, such as DNA methyltransferase inhibitors and histone deacetylase inhibitors, also has a pronounced effect on restoring or increasing MHC molecular expression [118–121]. Besides, reduction of MHC expression caused by autophagy can be another common tumor escape mechanism in a solid tumor. Autophagy inhibition recovers the MHC level of the tumor cell surface and promotes T cell activation [122]. NK cells acquire disinhibition when tumor cells decrease MHC molecular expression, and become activated when detecting ligands of activating receptors. Thus, exploiting the NK-involved antitumor mechanism can be a feasible and reasonable strategy to counteract tumor escape. A vaccine designed to induce antibodies that anchor MICA/B has been demonstrated to prevent tumor escape and enhance the function of tumor-reactive T cells and APCs (Fig. 3C b) [123]. Notably, this vaccine targeting MHC-I expressing tumors can be applied in clinical ACT as an inexpensive and effective “off-the-shelf” drug.

### Influence of T cell infiltration and tumor microenvironment on the efficacy of immunotherapy

**Fig. 4:**
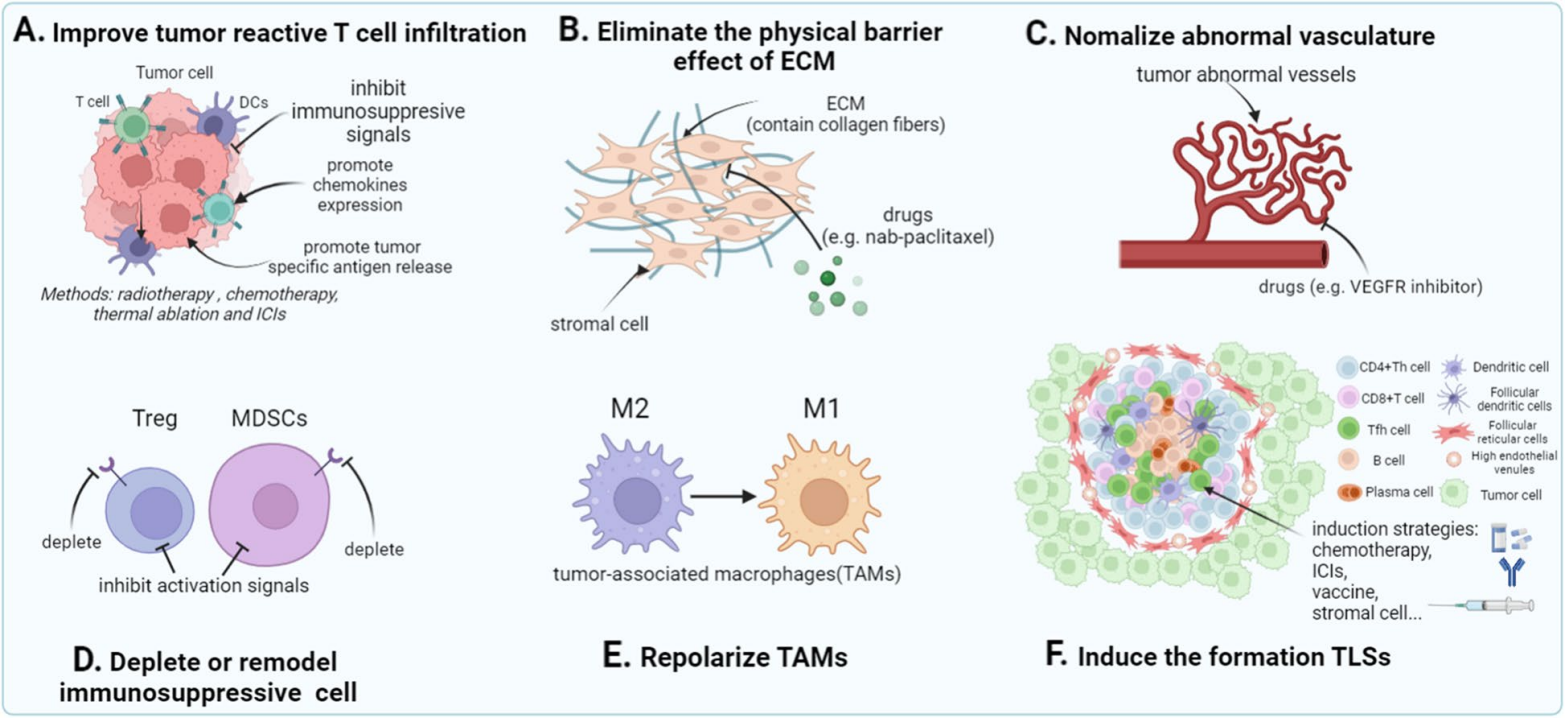
Strategies for promoting NRT cell tumor infiltration and modifying suppressive TME. **A** Improve tumor reactive T cell infiltration through inhibiting immunosuppressive signals, and promoting the expression of chemokines and the release of tumor specific antigens. **B** Eliminate the physical barrier effect of extracellular matrix(ECM) using drugs such as nab-paclitaxel. **C** Normalize abnormal vasculature by using VEGFR inhibitor. **D** Deplete immunosuppressive cells(Treg and MDSCs) and inhibit their activation signals. **E** Repolarize tumor-associated macrophages(TAM) from M2 towards M1. **F** Induce the formation of tertiary lymphoid structures(TLSs)(chemotherapy, ICIs, vaccine and stromal cell)

**Fig. 5:**
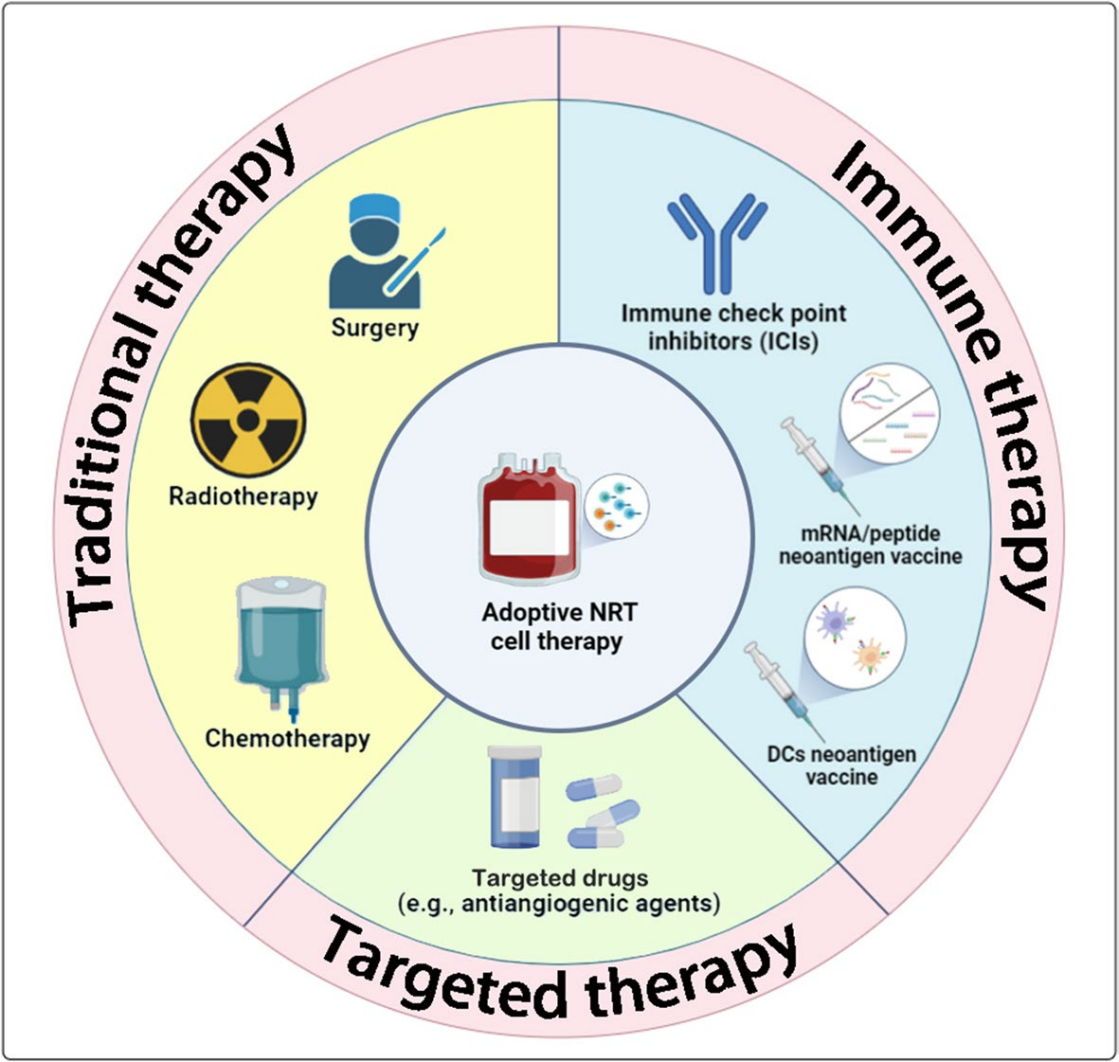
Feasible combinational therapy strategies of adoptive NRT cell therapy. Feasible combinational therapy strategies are shown in this figure. Adoptive NRT cell therapy can be combined with such strategies: immune therapy (immune check point inhibitors (ICIs), mRNA/peptide neoantigen vaccine, DC neoantigen vaccine), targeted drugs (e.g., antiangiogenic agents) and traditional therapy (surgery, radiotherapy and chemotherapy)

Although adoptive therapy of engineered T cells shows remarkable efficacy in clinical therapy, the interaction among malignant cells, immune cells, and other stromal components also requires deep exploration, which will offer feasible approaches to improving the infiltration and potency of T cells, preferably NRT. In some recent reviews, turning “cold tumors (immune-excluded and deserted tumors)” into “hot tumors (immune-inflamed tumors)” has become a research hot spot to strengthen immunotherapy efficacy [124, 125]. Compelling evidence proves that infiltration of antigen-specific T cells within tumors promotes favorable clinical outcomes. However, tumor-infiltrating T lymphocytes expressing high-level inhibitory receptors trigger downregulation of antitumor response, and low-level expression of chemokine receptors leads to poor T cell infiltration [126]. Strategies to improve T cell infiltration have been proposed to overcome these challenges (Fig. 4A). With radiotherapy and chemotherapy, tumor cells undergo immunogenic cell death (ICD), and release multiple cytokines and chemokines [127, 128]. Radiotherapy and thermal ablation can directly kill tumor cells or induce their apoptosis, as well as increase expression of MHC on the surface of the antigen presenting cells. [129, 130]. Compared with monotherapy, combination of NRT cell adoptive therapy or neoantigen vaccine with ICIs (Fig. 5) has been proven to achieve impressive outcomes [22, 24, 26]. Suppressive tumor microenvironment (TME) is composed of fibroblasts, immunosuppressive cells, abnormal proliferating vasculature and extracellular matrix, which may negatively impact host immune cell infiltration. Eliminating the physical barrier effect of extracellular matrix (ECM) by using ECM targeting agents can promote T cell infiltration into tumors (Fig. 4B), which has been addressed in the reviews of [131, 132]. The most common strategy for depleting the stroma is to use albumin-bound paclitaxel to facilitate T cell infiltration [133, 134]. Research showed that combinational therapy with nab-paclitaxel and gemcitabine or nab-paclitaxel and atezolizumab significantly improves tumor control and patient survival [135]. Aberrant growth of tumor vasculature will result in the formation of hypoxia and immunosuppressive TME [136]. Thus, normalizing the originally abnormal tumor vasculature via antiangiogenic agents (such as VEGFR inhibitor) and ICIs treatment will reduce hypoxia and remodel TME for a more favorable treating condition and potentiate antitumor immune cell activation (Fig. 4C) [136, 137]. Immune suppressive cells, such as Tregs, myeloid-derived suppressor cells (MDSCs) and tumor-associated macrophages (TAMs), are important study subjects in researches on overcoming resistance from TME. Depletion is the most common strategy for decreasing the quantity of both Tregs and MDSCs (Fig. 4D). Using anti-CCR4-antibody can deplete suppressive Treg cells in TILs and enhance antitumor-specific function of T cells [138]. For MDSCs, gemtuzumab can deplete intratumoral MDSCs, and the CXCR2 antagonist can block migration of MDSCs into tumors [139, 140]. Besides, targeting signaling pathway can also inhibit the proliferation and function of Tregs or MDSCs. Tregs can be inhibited by deleting transcription factor Blimp1 [141], while MDSCs can be inhibited by upregulating LXR expression [142], downregulating PEKR expression [143] or blocking CaMKK2 signal pathway [144]. In addition, another research hot spot is re-polarization of tumor-associated macrophages (TAM) from an anti-inflammatory (M2) phenotype to a pro-inflammatory (M1) one (Fig. 4E). This can be realized by using CpG-ODN [145] or non-coding RNAs as the immune regulator [146], or inhibiting the metabolism of lipid [147]. Tertiary lymphoid structures (TLSs) have a significant association with immune cell infiltration and cancer prognosis (Fig. 4F). In the two reviews of TLSs, the authors believed that solid tumors with more TLSs present a large quantity of effector memory T cells and cytotoxic T cells [148, 149]. Activated B cells in TLSs, except Bregs, can present antigens, stimulate activating signals, and secret cytokines to activate T cells and augment their antitumor function. Researches have demonstrated that the synergy work of B cells and T cells, together with the cooperation of humoral and cellular immunity, impacts the immune response and survival of patients [150, 151]. The number of TLSs is positively related to the efficacy of immunotherapy. And the induction of TLSs formation can be realized by applying ICIs [149, 152], vaccines [9], lymphoid chemokines or stromal cells [153, 154]. The abovementioned strategies strive to facilitate T cell infiltration, reinvigorate and augment the function of effector T cells, induce memory T cell generation, and eventually remodel adverse TME. These strategies can be performed by inhibiting immunosuppressive signals, increasing chemokine expression, removing barriers from TME, depleting or remodeling immunosuppressive cells, re-directing TAMs toward antitumor phenotype, and inducing formation of TLSs within the tumor. These measures dedicated to overcoming extracellular resistance can also improve the efficacy of adoptive NRT cell therapy in solid tumor treatment.

### Clinical Trials for NRT cell therapy

We have summarized the clinical trials of NRT cell adoptive therapy and other NRT cell-related immunotherapies. Twenty-six eligible researches are incorporated, among which six are on NRT cell adoptive therapy, one on NRT cell adoptive therapy plus neoantigen vaccine (Neovax), one on neoantigen dendritic cell vaccine (Neo-DCVac) plus NRT cell adoptive therapy, seven on NeoVax, two on Neovax plus ICIs, three on tumor infiltrating lymphocytes (TILs), one on TIL plus ICIs, two on Neo-DCVac, one on Neo-DCVac plus ICIs and two on ICIs monotherapy. Almost all these researches are in phase I or II, and the majority are applied in melanoma due to its high mutant frequency. We have found that patients receiving NRT cell adoptive therapy or neoantigen vaccine therapy combined with ICIs outperform those who only receive monotherapy. In the following paragraphs we will mainly introduce clinical trials of NRT cell therapy. Other researches of NRT cell-related immunotherapy will be listed in Table 2. Schemes of feasible NRT cell combinational therapy are shown in Fig. 5.

**Table 2:** Current NRT cell therapy and NRT cell relative clinical researches

**Identifier**	Year	Cancer	Stage	Phase	Patients	Intervention	Name	Targets or formulation	Primary outcome	NRT cell induction or response	Inference
**NCT01174121**	2014	cholangio-carcinoma		II	1	NRT		ERBB2IP-E805G CD4 + Th1 cell	SD	Vβ22 + ERBB2IP NRT cells exerted a major antitumor effect.	[155]
**NCT03171220**	2017	solid tumors	advanced		6	NRT			CR: 1; PR: 1; SD: 4; PFS (median): 8.6 M	De novo approach: less than 30% of the peptides can induce NRT cells; Peptide library approach: nearly 50%.	[27]
**NCT01174121**	2017	breast cancer	advanced (ER + HER2–)	II	1	NRT		mutSLC3A2, mutKIAA0368	CR	SLC3A2 reactivity and KIAA0368 reactivity were mediated by CD4 + and CD8 + T cells, respectively. 11 TCR clonotypes recognized the four neoantigens (SLC3A2, KIAA0368, CADPS2 and CTSB). 72.7% of the TCR of NRT cells can be detected in patient’s peripheral blood, lasting at least 17 months.	[26]
**ChiCTR1800017836**	2020	collecting duct carcinoma	advanced	I	1	NRT + Neovax		13 neoantigen peptides	SD	12 of 13 peptides can induce NRT cells. The frequency of mutant allele decreased after three months.	[28]
**NCT03199807**	2021	HCC	IV		1	NRT		KRAS-G12A, KRAS-G13D, PIK3CA-H1047L, IDH1-R132H	CR	The proportion of NRT cells in vivo reached a maximum of 4.49% in four cycle reinfusions.	[25]
**NCT03935893**	2022	solid tumor	advanced	II	1	NRT		KRAS-G12D TCR T cell	CR	Reinfused NRT cells represented approximately 2.4% of the total circulating T cells at 6 months.	[156]
**NCT01174121 and NCT03412877**	2022	solid tumor	advanced	II	13	NRT		TP53 TCR T cell	PR: 3; NR: 10	Percentage of NRT cells with a persistence of 6 weeks: 0.01%; NRT cells showed high level exhausted phenotype (PD-1:43%, TIM3: 33%, CD39: 93%) and low-level central memory phenotype (CD62L:5.02%).	[157]
**NCT03067493**	2022	primary HCC		II	23(10 vaccinated)	NRT + Neo-DCVac		peptide–loaded DC	SD: 5; PD: 5; DSF(median): 18.3 M	42.5% of the neoantigen peptides induced NRT cell response; 70% of patients had neoantigen-specific immune response.	[158]
**NCT02035956**	2017	melanoma	III-IV	I	13	NeoVax	IVAC MUTANOME	mRNA	SD: 8; PD: 5	60% of the neoantigen peptides induced NRT cell response; each patient developed NRT cells against at least 3 mutations; 57% of NRT cells were CD4 + T cell.	[159]
**NCT02287428**	2018	MGMT-UG	I/Ib	I	10 (2 withdrew)	NeoVax		peptides + poly-ICLC	PD: 8; PFS(median): 7.6 M; OR: 16.8 M	Two patients who did not receive dexamethasome generated NRT cell response towards neoantigens.	[160]
**NCT03480152**	2019	gastrointestinal cancer	advanced	I	5(1 PD)	NeoVax	mRNA-4650	mRNA	NR	15.7% of the neoantigen peptides induced NRT cell response; 59% of NRT cells were CD4 + T cells and 41% were CD8 + T cells.	[161]
**NCT03662815**	2019	solid tumors	advanced		24 (22 vaccinated, 21 finish five)	NeoVax	iNeo-Vac-P01	peptides + GM-CSF	SD: 15; PD: 6; PFS(median): 4.6 M; PFS(6): 27.3%; OS(12): 55.1%;	Nearly 80% of the peptides or peptide pools induced NRT cell response.	[162]
**NCT01461148**	2020	colorectal cancer	III/IV	I/IIa	22(19 finished)	NeoVax	FSP (TAF1B (-1), HT001 (-1) and AIM2 (-1)	peptides	SD: 3	50% of the patients had neoantigen-specific immune response. Each patient developed NRT cells against at least 1 peptide. Some frameshift peptide neoantigens could not induce CD8 + NRT cells.	[163]
**NCT02960230**	2020	diffuse midline glioma	HLA-A^*^02:01 + , H3.3K27 Mut		19	NeoVax		peptides + TT peptide + poly-ICLC	OS(median): 16.1 M, OS(12): 40%	Nearly 80% of the neoantigen peptides induced NRT cell response. Expansion of CD8 + NRT cells was associated with a better prognosis.	[164]
**ChiCTR1900020990**	2021	HCC	II-III	I	10	NeoVax		peptides + poly-ICLC	CR: 2; PD: 8; median RFS: 7.4 M;	Nearly 70% of the neoantigen peptides induced NRT cell response. 50% of the patients had neoantigen-specific immune response.	[165]
**NCT01970358**	2017	melanoma	IIIB/C, IVM1a/b	I	8 (6 vaccinated)	NeoVax + ICIs	ICI: pembrolizumab	peptides + poly-ICLC	CR: 2; SD: 6; PD: 2; PFS: 25 M;	47% of the neoantigen peptide pools induced NRT cell response. 20% of the neoantigen peptides induced CD4 + T cell response. The proportion of neoantigen stimulation was higher in MHC class II than in MHC class I.	[22]
**NCT02897765**	2020	melanoma, NSCLC, and TCC	III/IV		82(60 vaccinated)	NeoVax + ICIs	NeoVax: Neo-PV-01,ICI: Nivolumab	peptides + poly-ICLC	PFS (median): 23.5 M (melanoma), 8.5 M (NSCLC); 5.8 M (TCC). OS(1Y): 96% (melanoma), 83% (NSCLC), 67% (TCC)	52% of the neoantigen peptides induced NRT cell response in melanoma patients, 47% in NSCLC and 52% in bladder cancer patients. The average proportion of the immune response induced by neoantigen were 42% in CD4 + T cells and 24% in CD8 + T cells.	[24]
**NCT01807182**	2018	melanoma	III-IV	II	1	TIL		BRAF^V600E^	CR	Neoantigen peptides only stimulated CD4 + T cell response.	[166]
**NCT02278887**	2020	melanoma	IIIc/IV	I	10	TIL			CR: 2; PR: 3; SD: 1; PD: 4	NRT cells can be detected in 66.7% of the patients with TIL infusion; The frequency of NRT cells responses peaked between 3 and 9 months.	[167]
**NCT00937625**	2021	melanoma	advanced	I	26	TIL			CR: 5; PR: 6; SD: 10; PD: 5	NRT cells can be detected in 69.2% of the patients with TIL infusion; 3.4% of the neoantigen peptides in TIL or peripheral blood induced NRT cell response. The median proportion of CD8 + T NRT cells was 0.63%.	[168]
**NCT03215810**	2020	NSCLC	IV	I	20(16 vaccinated)	TIL + ICIs	ICI: nivolumab		CR: 2; PR: 2; SD: 2; PD: 1	72.2% of the patients had neoantigen-specific immune response. The majority of TILs were terminally differentiated and only small subsets were in the stem-like state.	[169]
**NCT00683670**	2013	melanoma	IV	I	7	Neo-DCVac			CR: 1; PR: 2; PD: 4	DC vaccine augmented NRT cell response and broadened neoantigen-reactive TCR repertoire. Only three neoantigens with the highest binding affinity can induce NRT cell response in each patient.	[170, 171]
**NCT02956551**	2020	lung cancer	IIIc/IV	I	18(12 vaccinated)	Neo-DCVac			SD: 9; PD: 3; PFS(median): 5.5 M; OS(median):7.9 M	Nearly 62% of the neoantigen peptides induced NRT cell response on average. The percentage of CD8 + NRT cells was 21.69% and that of CD4 + NRT cells was 43.72% on average.	[172]
**NCT01132014**	2017	ovarian cancer	IIIb-IV	I	25	Neo-DCVac + ICIs	ICI: bevacizumab	DC pulsed with oxidized autologous whole-tumor cell lysate	SD: 16; PD: 9; PFS(24 months): 25; OS(2Y, responder): 100%, OS(2Y,no responder): 25%;	Six patients who received DC vaccine generated NRT cells targeting at least one neoantigen. CD8 + NRT cells induced by DC vaccine were polyfunctional.	[173]
**NCT02108652**	2014	UBC	IV	II	24	ICI	Atezolizumab		Early-stage NART expansion and activation are associated with response to ICB.	Increased NRT cell response can be observed between pre-treatment to three weeks post-treatment of ICI. The phenotypes of NRT cells with PD1 + Ki67 + effector and increased CD39 levels showed positively relevant to clinical response.	[174]
**NCT01903993**	2019	NSCLC	IIIb-IV	II	14	ICI	Atezolizumab		Neoantigen-specific T cells phenotype change: differentiated effector phenotype, memory-like phenotypic (PD).	Patients who responded to ICI therapy showed increased frequency of NRT cells and differentiated effector phenotype of T cells.	[175]

*UBC* Urothelial bladder cancer, *MGMT-UG* MGMT-unmethylated glioblastoma, *NSCLC* Non-small cell lung cancer, *TCC* Urothelial carcinoma, *HCC* Hepatocellular carcinoma, *NRT* Neoantigen reactive T cell, *NeoVax* Neoantigen vaccine, *Neo-DCVac* Neoantigen dendritic cells vaccine, *ICI* Immune checkpoint inhibitor, *TIL* tumor infiltrating lymphocyte, *FSP* Frameshift peptide, *GM-CSF* Granulocyte–macrophage colony stimulating factor, *CR* complete response, *PR* partial response, *SD* Stable disease, *PD* Progressive disease, *NR* No response, *OS* Overall survival, *PFS* Progression-free survival, *DFS* Disease-free survival, *RFS* Relapse free progression, *Y* year (s), *M* Month (s))

The majority of traditional engineered TCR T cells are designed to target tumor-associated antigens (TAAs) while relatively few teams have conducted researches on neoantigens [89, 176]. It is difficult for these T cells to eliminate tumor cells thoroughly due to their heterogeneity, and patients also show limited clinical responses or even suffer autoimmune diseases caused by the “off-target” effect [177–180]. Rosenberg’s team is devoted to exploring NRT cell populations targeting shared antigens and developing engineered neoantigen-targeted TCR T cells as “off-the-shelf” products. Their first NRT cell therapy case was a metastatic cholangiocarcinoma patient who received ERBB2IP-targeted CD4 + T cell therapy and achieved disease stability for more than one year after twice reinfusions [155]. KRAS-G12D-targeted NRT cells in gastrointestinal cancers have also been screened [181, 182]. Recently, a case report showed that a pancreatic cancer patient who received KRAS-G12D NRT cell therapy obtained a 6-month partial objective response accompanied by long-term existence of effector T cells [156]. Besides, twelve patients in two clinical trials who harbored TP53 mutation received NRT cell therapy. Among them, two patients exhibited a partial response, and another patient with chemo-refractory breast cancer realized tumor regression that lasted for at least six months [157]. Another research team conducted two pioneering clinical trials, showing the value of transferring NRT cells in treating advanced and refractory solid tumors. In the first study, researchers compared the therapeutic effects of two sources of neoantigens-de novo and shared library. In three patients treated with NRT cells manufactured by the de novo pattern, the overall response rate of T cells to neoantigens was lower than 34%. However, using a shared neoantigen library could significantly increase the efficiency and accuracy of hot spot mutation identification. Six patients using NRT cells made by this pattern achieved one CR, one PR, and four SD [27]. In the second study, a patient with advanced hepatocellular carcinoma (HCC) received NRT cell therapy combined with radiotherapy and ICI therapy, and realized partial response and complete regression in the new lesion [25]. This study completed the first comprehensive NRT cell therapy in an advanced HCC patient who benefited from prolonged survival without severe side effects. In addition, other studies have also shown favorable clinical results of NRT cell therapy. Nikolaos Zacharakis et al. presented a case of a breast cancer patient with complete regression after reinfusion of NRT cells targeting four individual somatic mutations combined with ICIs [26]. Our team also reported a case of a collecting duct carcinoma (CDC) patient who obtained SD with decreased tumor loads and regression of metastatic lesions after administration of NRT immunotherapy [28]. More than 92% of the neoantigens in this research could fully stimulate reactive T cells in PBMC. The activation proportion of NRT elevated from 1.92% to 7.92%. The latest phase II clinical trial used NRT cell therapy combined with DC neoantigen vaccine, chemotherapy, radiofrequency ablation, and ICIs to treat hepatocellular carcinoma [158]. Fifty percent of the patients obtained disease stability without relapse for two years. Other patients failed to respond due to depletion of tumor neoantigen and generation of new neoantigen epitope. The overall safety of adoptive NRT cell therapy is good and no prominently serious adverse events have been observed. Only two among the seven studies of NRT cell therapy reported minor adverse reactions of grade 1–2 [27, 157]. These results demonstrate that this therapy is feasible and safe for the activation of autologous NRT cells to eliminate tumor cells.

Furthermore, some recruiting studies on NRT cell therapy and engineering TCR neoantigen T cells are listed in Table 3. All these researches aim at solid tumors, including three in phase I, six in phase I/II, and two in phase II clinical trials. Four studies use shared NRT cells, while two use de novo NRT cells. Six studies apply NRT cells combined with ICIs, one of which also adds CDX-1140, a monoclonal antibody targeting CD40. The feasibility and safety of these researches need to be confirmed by the publication of the latest results.

**Table 3:** Recruiting researches of NRT cell and engineering TCR T cell therapy

**Identifier**	Posted Year	Cancer	Intervention	Target	Phase	Combination	Country	Status
**NCT03190941**	2017	gastrointestinal and pancreatic cancer	engineering TCR-T cell	HLA-A^*^11:01, KRAS G12V	I/II	none	United States	recruiting
**NCT03745326**	2018	gastrointestinal and pancreatic cancer	engineering TCR-T cell	HLA-A^*^02:01,KRAS-G12D	I/II	none	United States	recruiting
**NCT03412877**	2018	solid tumor	engineering TCR-T cell	unknown	II	Pembrolizumab	United States	recruiting
**NCT04102436**	2019	solid tumor	engineering TCR-T cell	unknown	II	none		recruiting
**NCT03970382**	2019	solid tumor	engineering TCR-T cell	unknown	I	Nivolumab	United States	Suspended
**NCT04032847**	2019	NSCLC	NRT cells(ATL001)	mutiple neoantigens	I/II	Pembrolizumab	United Kingdom	recruiting
**NCT03997474**	2019	melanoma	NRT cells(ATL001)	mutiple neoantigens	I/II	Nivolumab	United Kingdom	recruiting
**NCT04146298**	2019	pancreatic cancer	NRT cells	HLA-A^*^11:01,KRAS G12V	I/II	Anti-PD-1 monoclonal antibody	China	recruiting
**NCT04520711**	2020	malignant epithelial cancer	engineering TCR-T cell	unknown	I	CDX-1140 + Pembrolizumab	United States	recruiting
**NCT05194735**	2022	solid tumor	engineering TCR-T cell	unknown	I/II	none	United States	recruiting
**NCT05478837**	2022	diffuse midline glioma	engineering TCR-T cell	HLA-A^*^0201, H3.3K27M	I	none	United States	not yet recruiting

The studies above show that NRT cell therapy realizes favorable tumor regression and long-term antitumor effect, especially for patients of end-stage melanoma or refractory solid tumors, in a more individualized or “off-the-shelf” way. However, due to the inaccuracy of prediction algorithms or suppression of TME, the overall response to NRT cell therapy is limited. In some cases, shared antigens are not included in the top alternative neoantigens, which means driver gene peptides are not the optimal targets in some cancer patients. The efficacy of adoptive NRT cell therapy will be improved by both traditional therapy and other immunotherapies, which can broaden the repertoire and augment the function of autologous NRT cells.

### Limitations of NRT cell therapy

Although adoptive NRT cell therapy has superiority in effectiveness and safety in advanced tumor treatment, it still has some limitations. Under the pressure of immune editing, the consequence of the tumor cell evolution is that the quantity of cancer neoantigens originating from driver mutation will decrease while the number of those deriving from passenger mutation will increase. Thus, NRT cells designed to target single driver gene mutations (e.g., KRAS, TP53) fail to achieve complete regression of primary tumors. Moreover, unlike driver mutation-derived neoantigens, passenger mutation-derived neoantigens are different in each patient, suggesting that the cell products for each patient need to be tailored. Besides, the deficiency of predicting platforms leads to the deviation of neoantigen prediction and dissatisfactory treatment efficacy. Compared with the neoantigen vaccine, adoptive NRT cell therapy targets fewer neoantigens and induces a narrower breadth of the immune response [5, 183], and the process of NRT cell manufacturing is complicated, time-consuming and costly. Existent evidence has shown that ex vivo cultivation will increase the proportion of the terminal differential phenotype of T cells and reduce activity and proliferation of NRT cells [184–186], whereas neoantigen vaccine will not result in these problems due to it induces NRT cells activation directly in vivo. Furthermore, potential cytokine release syndrome requires additional attention in NRT cell-based therapy, and IL-1 and IL-6 receptor antagonists or blockades are needed to cope with this problem [187, 188].

## Conclusion

The discovery of neoantigens boosts the development of individually-tailored immunotherapy, including adoptive therapy with T cells. With more efficient and precise therapeutic potency, T cells stimulated by neoantigens exhibit powerful antitumor capability. Based on bio-information technology, T cell screening and engineering techniques, modified NRT cells can be implemented more economically and conveniently. Although the feasibility and safety of NRT-related immunotherapy have been verified, the majority of researches are still in the initial stage, and the overall treatment results are unsatisfactory. Improved methods have been proposed to meet the urgent demand for improvement of therapy effectiveness and development of novel platforms as well as of multiple-drug combinatorial therapy. Current challenges to adoptive NRT cell therapy are the high cost and difficulty in realizing individualization, which renders industrial mass production unlikely and needs to be solved by future technological innovation. However, generally speaking, we are convinced that NRT cell-based immunotherapy has the effectiveness and safety to realize enduring tumor elimination and significantly prolonged survival that benefit patients with advanced solid tumors.

## Data Availability

Not applicable.

## Abbreviation

ACT: Adoptive cell therapy
AICD: Activation-induced T cell death
APC: Antigen presenting cell
CAR-T: Chimeric antigen receptor-T cell
CDC: Collecting duct carcinoma
CR: Complete response
DC: Dendritic cell
ECM: Extracellular matrix
HCC: Hepatocellular carcinoma
ICD: Immunogenic cell death
ICI: Immune checkpoint inhibitor
MDSC: Myeloid-derived suppressor cell
MHC: Major histocompatibility complex
MICA/B: MHC class I polypeptide–related sequence A/B
Neo-DCVac: Neoantigen dendritic cell vaccine
NeoVax: Neoantigen vaccine
NRT cells: Neoantigen-reactive T cells
PBMC: Peripheral blood mononuclear cell
PR: Partial response
REP: Rapid expansion protocol
SD: Stable disease
TAA: Tumor associated antigen
TAM: Tumor-associated macrophages
TCR: T cell receptor
TIL: Tumor infiltrating lymphocyte
TLS: Tertiary lymphoid structure
TME: Tumor microenvironment
TSA: Tumor specific antigen
VEGFR: Vascular endothelial growth factor receptor
WES: Whole exome sequencing
